# Contrasting effects of glutamate and branched-chain amino acid metabolism on acid tolerance in a *Castellaniella* isolate from acidic groundwater

**DOI:** 10.1128/aem.01942-25

**Published:** 2026-01-30

**Authors:** Jennifer L. Goff, Konnor L. Durrence, Michael P. Thorgersen, Valentine V. Trotter, Yan Chen, Suzanne M. Kosina, Audrey L. W. Wang, Farris L. Poole, Trent R. Northen, Christopher J. Petzold, Adam M. Deutschbauer, Michael W. W. Adams

**Affiliations:** 1 Department of Biochemistry and Molecular Biology, University of Georgia 174518, Athens, Georgia, USA; 2 Department of Chemistry, SUNY College of Environmental Science and Forestry 539343, Syracuse, New York, USA; 3 Environmental Genomics and Systems Biology Division, E.O. Lawrence Berkeley National Laboratory 1666, Berkeley, California, USA; 4 Biological Systems and Engineering Division, E.O. Lawrence Berkeley National Laboratory 1666, Berkeley, California, USA; 5 Joint Genome Institute, E.O. Lawrence Berkeley National Laboratory 229279, Berkeley, California, USA; 6 Department of Plant and Microbial Biology, University of California 118549, Berkeley, California, USA; Colorado School of Mines, Golden, Colorado, USA

**Keywords:** heavy metals, denitrification, acid tolerance

## Abstract

Groundwater acidification co-occurring with nitrate pollution is a common, global environmental health hazard. Denitrifying bacteria have been leveraged for the *in situ* removal of nitrate in groundwater. However, co-existing stressors—such as low pH—reduce the efficacy of biological removal processes. *Castellaniella* sp. str. MT123 is a complete denitrifier that was isolated from acidic, nitrate-contaminated groundwater. The strain grows robustly by nitrate respiration at pH < 6.0, completely reducing nitrate to dinitrogen gas. Genomic analyses of MT123 revealed few previously characterized acid tolerance genes. Thus, we utilized a combination of proteomics, metabolomics, and competitive mutant fitness to characterize the genetic mechanisms of MT123 acclimation to growth under mildly acidic conditions. We found that glutamate accumulation is critical in the acid acclimation of MT123, possibly through consumption of intracellular protons via glutamate decarboxylation to GABA. This is despite the fact that MT123 lacks the canonical glutamate decarboxylase-glutamate/GABA antiporter system implicated in acid tolerance in other bacteria. In contrast, branched-chain amino acid (BCAA) accumulation was detrimental to cell growth at lower pHs, possibly through indirect mechanisms impacting the cellular glutamate pool. Genetic analysis previously linked MT123 to a population of *Castellaniella* that bloomed—concurrent to nitrate removal—during a biostimulation effort to reduce groundwater nitrate concentrations at MT123’s location of origin. Thus, our analyses provide novel insight into mechanisms of acclimation to acidic conditions in a strain with significant potential for nitrate bioremediation.

**IMPORTANCE:**

Nitrate pollution in groundwater is a major threat to both environmental and human health. This nitrate pollution can come from a variety of sources, including farm fertilizers, sewage, animal waste, septic systems, and industrial discharge. Bacteria known as “denitrifiers” can convert this nitrate into harmless nitrogen gas, a process known as “denitrification.” Denitrifiers can be used to clean up nitrate-contaminated groundwater. However, their ability to do this can be disrupted by changing environmental conditions. For example, groundwater that is polluted with nitrate is often acidic. Acidic conditions make it challenging for denitrifiers to survive, which results in less conversion of nitrate to nitrogen gas. In this study, we investigated how one denitrifying bacterium—originating from acidic, nitrate-contaminated groundwater—can cope with acidic conditions.

## INTRODUCTION

Nitrate pollution in groundwater is a major environmental health hazard, globally (1). This nitrate originates from sewage runoff, livestock facilities, septic systems, industrial wastewater, agricultural fertilization, and atmospheric deposition (2). At concentrations as low as 50 mg/L, nitrate ingestion can cause methemoglobinemia in neonates and young children (2). Groundwater nitrate concentrations exceeding this threshold have been reported across the world (3).

Denitrifying bacteria have been leveraged for nitrate removal in groundwater (4). Biostimulation of contaminated groundwater with organic carbon promotes the rapid proliferation of denitrifying bacteria that reduce nitrate to dinitrogen gas (5–8). However, these efforts are challenged by additional dynamic physiochemical conditions to which these bacteria are responsive. For example, co-occurring contaminants, such as heavy metals, can inhibit the activities of enzymes in the denitrification pathway (9). Additionally, acidification is a common co-occurring problem in nitrate-contaminated groundwater (10), where the presence of the nitrate can itself promote acidification (11–15). Mildly acidic conditions with pHs lower than 6.0 to 6.5 inhibit the activity of nitrous oxide reductase NosZ (16–18) and decrease activity of nitrite reductases (19). Inhibition of individual steps within the denitrification pathway leads to the undesirable consequence of partial denitrification, resulting in the release of reactive nitrogen oxides, such as nitrite, nitric oxide, and nitrous oxide (20, 21). Thus, it is critical to understand how denitrifiers respond to these common co-occurring environmental stressors.

The Oak Ridge Reservation (ORR, Oak Ridge, TN, USA) is a well-characterized experimental site for examining the ecological impacts of legacy industrial waste (22). Previously, industrial waste generated from nuclear processing operations was dumped into four unlined waste ponds at ORR, known as the S-3 ponds. The soils and groundwater near the point source are highly acidic due to high concentrations of uranium nitrate in the industrial waste (22). *Castellaniella* is a genus of facultatively anaerobic, denitrifying bacteria (23) that is highly abundant in the contaminated soils and groundwater at the ORR (24). Native *Castellaniella* strains were important for nitrate remediation in contaminated ORR soils (25), and *Castellaniella* was the dominant genus that bloomed during a successful biostimulation campaign, which decreased local nitrate concentrations (25). In an earlier analysis, we reported that this genus is commonly found in global anthropogenically impacted sites, including contaminated soil and water, wastewater treatment plants, and the built environment (24). Previous studies have also linked *Castellaniella* to nitrate removal in coking wastewater (26), smelting wastewater (27), sewage (28), granular sludge (29), and agricultural soils (30). Thus, *Castellaniella* species can sustain nitrate removal across diverse—and often physiologically stressful—environments, highlighting their potential as versatile and robust candidates for bioremediation applications.

We isolated *Castellaniella* sp. str. MT123 from highly contaminated ORR groundwater taken from within the S-3 ponds’ contamination plume (31). This strain is a representative of a highly abundant and persistent OTU (operational taxonomic unit) that was first reported at the site following biostimulation efforts in the early 2000s (24). In a sediment core collected after biostimulation with ethanol and bicarbonate, this OTU was reported at 70% relative abundance (25). MT123 grows optimally under the mildly acidic conditions of pH 5.5. The strain is a complete denitrifier, retaining this activity even at lower pHs (24). We hypothesized that this strain must have robust mechanisms for maintaining intracellular pH during growth under mildly acidic conditions. Here, we characterized the mechanisms of pH homeostasis in MT123. RB-TnSeq analysis was used to identify single-gene mutants with decreased fitness at pH 5.5 compared to 7.5. We paired these results with proteomic and metabolomic analyses to characterize systems-level changes occurring during mildly acidic growth conditions. Our results show contrasting roles of glutamate and branched-chain amino acids (BCAA) in the cellular response to mildly acidic growth conditions.

## MATERIALS AND METHODS

### Media and culture conditions

As described previously, *Castellaniella* sp. str. MT123 was isolated from groundwater samples taken from the contaminated well FW104 near the former S-3 waste ponds at the ORR (31). Glycerol stocks of MT123 were streaked on R2A agar plates (per liter: 0.5 g casein hydrolysate, 0.5 g dextrose, 0.5 g soluble starch, 0.5 g yeast extract, 0.3 g dipotassium phosphate, 0.3 g sodium pyruvate, 0.25 g proteose peptone, 0.25 g meat peptone, 15 g agar, and 0.05 g MgSO_4_ ∙ 7H_2_O). For precultures, 5 mL of R2A broth was inoculated with five individual colonies from the R2A agar plates and grown at 30°C aerobically with shaking (200 rpm). For experimental work, the overnight preculture was inoculated in modified UGA medium. The modified UGA medium contained, per liter, 0.6 g NaH_2_PO_4_, 20 mL of 1 M sodium lactate, 40 mL of a 25× UGA salts stock solution, 1 mL of a 1,000× DL vitamins stock, and 10 mL of a 100× DL minerals stock. The 25× UGA salts stock solution contained, per liter, 250 mg NaCl, 367 mg CaCl_2_∙ 2H_2_O, 12.32 g MgSO_4_ ∙ 7H_2_O, 2.5 g KCl, and 5 g NH_4_Cl. The 1,000× DL vitamins stock and the 100× DL minerals stock recipes are described previously (32). For any cultures grown in anaerobic conditions, the medium also contained 10 mL of 1 M NaNO_3_ per liter with an 80%/20% N_2_/CO_2_ headspace. The pH of the medium was adjusted, as indicated (4.5 to 7.5), with HCl or NaOH. Different buffers were used based on the desired culture pH. For cultures grown in pH < 7, we added 7.8 g of MES buffer per liter. For cultures grown in pH ≥ 7, we added 8.4 g of MOPs per liter.

### Growth phenotyping experiments

Growth experiments were performed in 100-well plates using a Bioscreen incubating plate reader (Growth Curves Ltd.), with a final volume of 400 µL of modified UGA medium. For growth under denitrifying conditions, the Bioscreen was placed in an anaerobic chamber under a 78% N_2_/20% CO_2_/2% H_2_ headspace. For the amino acid additions, all amino acids were added at 1 mM concentrations. The Bioscreen monitored growth by optical density measurements at 600 nm (OD_600_) once every hour. The Bioscreen was set to shake cultures continuously at low amplitude throughout each growth experiment while holding the temperature at 30°C.

### Genomic analysis

The MT123 genome was published previously (24) and is available under BioProject number PRJNA1100609 at NCBI. The protein annotations used for this study are described in Table S1. Between the time of analysis and writing of this report, the completed version of the MT123 genome described above became publicly available. Table S2 relates the locus tags used for this study to the current version of the MT123 genome. Proteins previously identified as being involved in acid tolerance in *Escherichia coli* K-12 or *Helicobacter pylori* strain J99 were utilized to perform a BLASTp search against the MT123 genome to identify homologs (33). Protein sequences were retrieved from UniProt.

### RB-TnSeq experiments

The RB-TnSeq mutant library for MT123 was constructed by insertion of a barcoded mariner transposon as described previously (34). Expanded methodology can be found in the supplemental material. The individual mutants are mapped to the MT123 genome based on the annotations provided in Table S1. Table S2 relates the locus tags used for this study to the current version of the MT123 genome.

A glycerol stock (~1 mL) of the MT123 mutant library was inoculated into 100 mL of R2A medium containing 10 μg/mL kanamycin and grown aerobically. These precultures were grown at 30°C and shaken at 200 rpm. Precultures were grown to approximately mid-log phase (OD_600_ = 0.5), and 1 mL of a 1.0 OD_600_ equivalent of preculture was recovered for sequencing to use as the reference library for all challenge condition cultures. Samples were spun in a microcentrifuge at 12,000 × *g* for 3 min. The supernatant was removed, and cell pellets were stored at −80°C for sequencing. Additionally, 1 mL of the 0.5 OD_600_ equivalent preculture was inoculated into 10 mL of the experimental medium.

Experimental cultures were grown in five replicates in the modified UGA medium described above using four different challenge conditions: aerobic (i.e., normal atmospheric conditions) at pH 5.5, aerobic at pH 7.5, anaerobic at pH 5.5, and anaerobic at pH 7.5. Experimental cultures were grown at 30°C. The anaerobic cultures were grown in sealed Hungate tubes with an 80%/20% N_2_/CO_2_ headspace. Aerobic cultures were grown in capped test tubes, shaken at 200 rpm. Experimental cultures were recovered at approximately mid-log phase (OD_600_ ≈ 0.5), with 1 mL corresponding to 1.0 OD_600_ equivalent collected and centrifuged at 12,000 × *g* for 3 min. The supernatant was removed, and cell pellets were stored at −80°C prior to genomic DNA extraction, PCR amplification of the DNA barcodes, and barcode sequencing (BarSeq) (34).

### RB-TnSeq data processing

We calculated gene fitness scores using a previously described approach (34). Averages of strain fitness values for each challenge condition were first calculated to determine a gene fitness value, after which several *Δfitness* values of interest were calculated. *Δfitness* values were calculated by subtracting the gene fitness value of the base condition (pH 7.5) from the challenge condition (pH 5.5). Genes with a large positive (*Δfitness* > 1) or large negative (*Δfitness* < −1) change in fitness value were selected for further analysis (35).

### Growth conditions for proteomic and metabolomic analyses

Five individual colonies of wild-type MT123 were inoculated from an R2A agar plate into 10 mL of R2A growth medium, which was then grown overnight as described above. A 40-fold dilution of the preculture was performed into 50 mL of modified UGA medium, as described above. Cultures for proteomic analyses were performed in triplicate, and cultures for metabolomic analyses were performed in sextuplicate. These experiments utilized the same four challenge growth conditions as those described above for the RB-TnSeq analysis. All experimental growth media were inoculated with 500 μL of preculture. The anaerobic experimental cultures were grown in sealed 100 mL serum bottles with an 80%/20% N_2_/CO_2_ headspace. Aerobic experimental cultures were grown in 250 mL Erlenmeyer flasks and shaken at 200 rpm. All cultures were grown at 30°C. When the cultures reached an OD_600_ of 0.5, each culture was centrifuged at 4,000 rpm for 5 min at 25°C. The supernatant was removed, and the pellet was washed with 5 mL of a pH 7.4 PBS buffer (per liter: 0.8 g NaCl, 0.02 g KCl, 0.144 g Na_2_HPO_4_, 0.024 g KH_2_PO_4_). Washed cell pellets were stored at −80°C prior to metabolomic and proteomic analyses.

### Proteomic analysis

Expanded methodology for the proteomic analyses can be found in the supplemental material. Briefly, protein was extracted from cell pellets, and tryptic peptides were prepared following the established proteomic sample preparation protocol (36). The resulting peptide samples were analyzed on an Agilent 1290 UHPLC system coupled to a Thermo Scientific Orbitrap Exploris 480 mass spectrometer for discovery proteomics (37). Briefly, peptide samples were loaded onto an Ascentis ES-C18 Column (Sigma-Aldrich, USA) for chromatographic separations. Eluting peptides were introduced to the mass spectrometer operating in positive-ion mode and were measured in data-independent acquisition (DIA) mode. DIA raw data files were analyzed using the integrated software suite DIA-NN (38). The databases used in the DIA-NN search (library-free mode) consisted of the protein FASTA sequences based on the MT123 genome annotations provided in Table S1, plus the protein sequences of common proteomic contaminants. Table S2 relates the locus tags used for this study to the current version of the MT123 genome. The output main DIA-NN reports were filtered with a global false discovery rate set at 0.01 (FDR ≤ 0.01) applied at both the precursor and protein group levels. The Top 3 method, which is the average MS signal response of the three most intense tryptic peptides of each identified protein, was used to quantify proteins in the samples (39, 40). A Welch’s *t*-test was used to compare peptide abundances between pH 5.5 and 7.5, using the Benjamini-Hochberg procedure to control the false discovery rate. A significant fold change was defined as |Log2FC| ≥ 1 and an adjusted *P*-value < 0.05. Clusters of Orthologous Genes (COGs) categories were assigned to the MT123 proteome using reCOGnizer (41).

### Metabolomic analysis

Frozen cell pellets were resuspended in 500 µL water, frozen at −80°C, lyophilized, and then homogenized with 3.2 mm stainless steel beads (BioSpec Products) in a Mini Bead Beater (BioSpec Products) three times for 5 s each, with 10 s of cooling between runs. The homogenized cell material was stored at −80°C. On the day of LC-MS/MS analysis, samples were resuspended in 150 µL of methanol containing internal standard mix (Table S4). The extracts were vortexed twice for 10 s, bath sonicated in ice water for 15 min, centrifuged (10,000 rcf, 5 min, 10°C), and then the supernatant was filtered through 0.22 µm PVDF microcentrifuge filter (10,000 rcf, 5 min, 10°C). Filtrate was transferred to amber glass LC-MS/MS vials for analysis. Briefly, metabolites were analyzed by both reverse-phase chromatography and hydrophilic interaction chromatography, each followed by tandem mass spectrometry analysis. Metabolite separations were performed using an Agilent 1290 LC stack and detected using a Thermo Q Extractive hybrid quadrupole-Orbitrap mass spectrometer. LC-MS/MS parameters are provided in Table S4. Internal and external standards as well as extraction and injection controls were used for quality control purposes.

### Metabolomic data processing

Raw data files are available in MZML format through https://gnps2.org/status?task=29f23392a1414aa2a75002938e2fecec. Metabolites were annotated using Metatlas (https://github.com/biorack/metatlas) (42) to compare m/z, retention time, and fragmentation spectra from samples to reference standards analyzed using the same methods. Annotations are provided in Table S3. To identify significant changes in metabolome abundance, a Welch’s *t*-test was performed to compare metabolite abundances at pH 5.5 and pH 7.5 under both growth conditions. All *P*-values were corrected for multiple comparisons using the Benjamini-Hochberg method. A significant fold change was defined as an adjusted *P*-value < 0.05.

## RESULTS

### MT123 grows optimally under mildly acidic conditions

**Fig 1:**
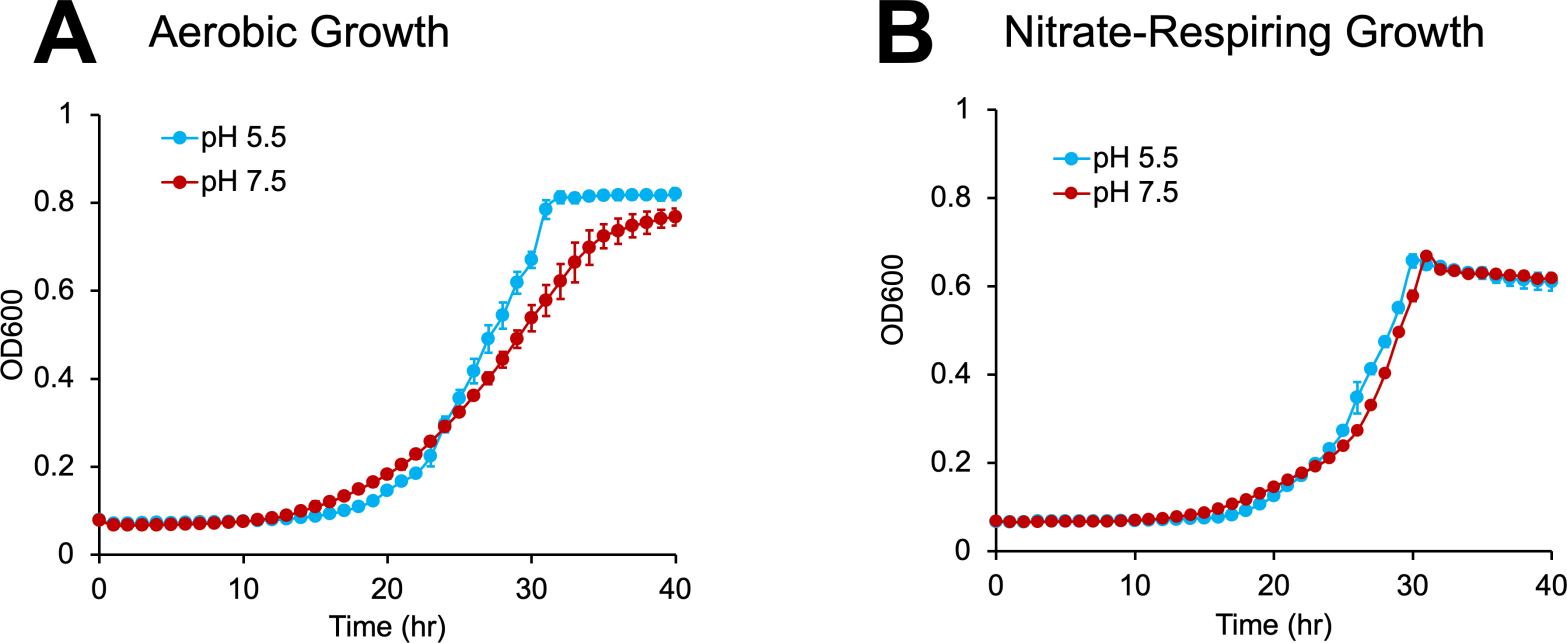
Growth of MT123 at pH 5.5 and 7.5 (legend shown on figure) under aerobic (**A**) and denitrifying (**B**) conditions. Data points represent the average of triplicate experiments. Error bars represent ±SD.

Concentrations of dissolved oxygen in groundwater can fluctuate over seasonal and storm-event timescales (43, 44). Thus, we first examined the growth of MT123 under both aerobic (i.e., normal atmospheric conditions) and denitrifying growth conditions at both neutral (pH 7.5) and mildly acidic (pH 5.5) pHs. Across the ORR subsurface, groundwater samples have been reported with a wide range of pH values, from 3 to 10.5 (45). A pH of 5.5 was chosen for this study because it reflects the pH of ORR groundwater well FW104, the isolation site of MT123. Consistent with our prior findings (24), under aerobic growth conditions, MT123 exhibited a higher maximal growth rate and slightly higher carrying capacity at pH 5.5 relative to 7.5 (Fig. 1A). At pH 5.5, a greater proportion of the denitrification intermediate nitrite exists in the form of free nitrous acid (FNA), a potent protonophore (46, 47) that is inhibitory to bacteria (48). Nonetheless, MT123 exhibits robust growth at pH 5.5 (Fig. 1B). Thus, we sought to understand the genetic mechanisms underlying MT123’s resilience under mildly acidic conditions.

### Genomic analysis of MT123 reveals a lack of classic acid tolerance systems

**Fig 2:**
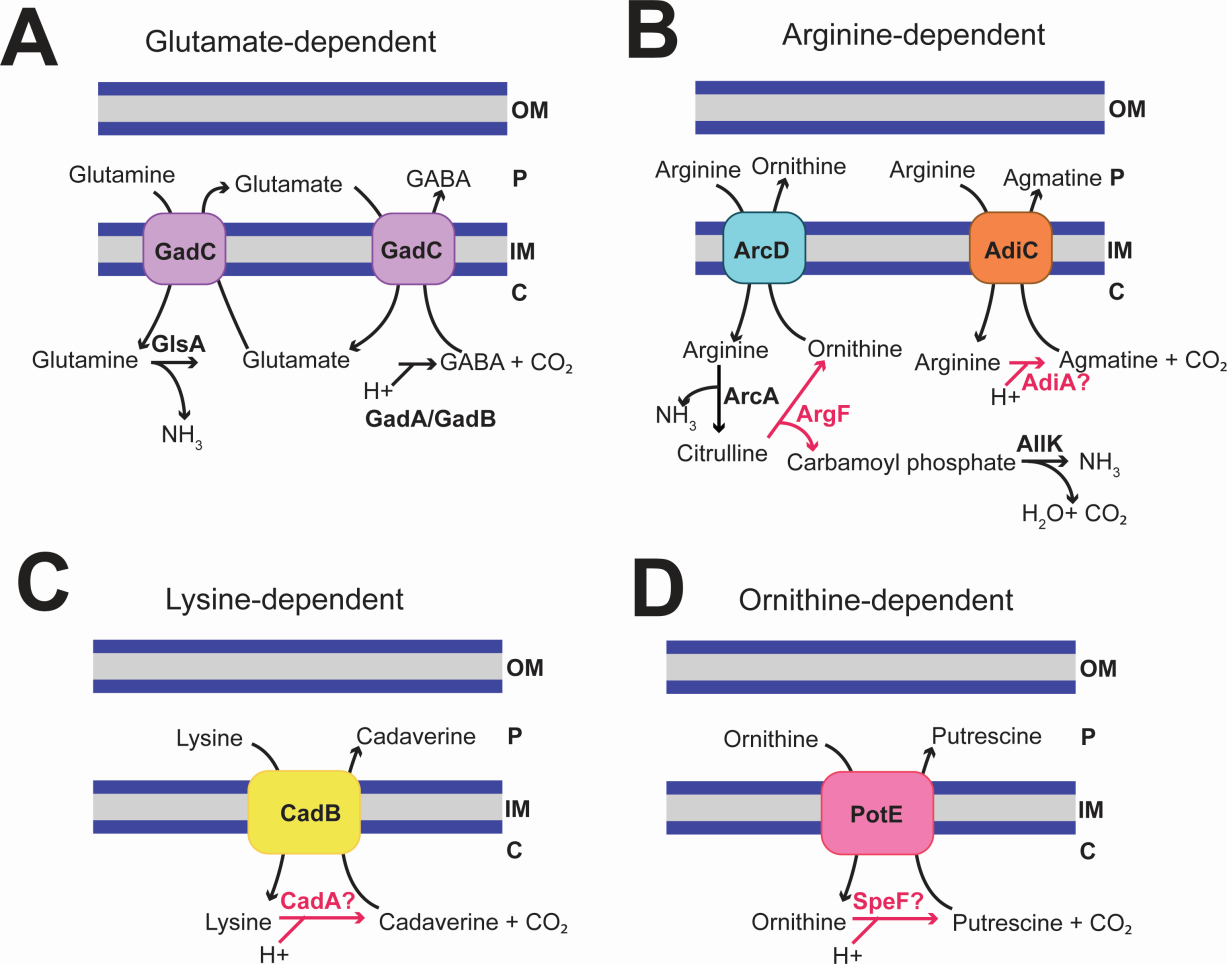
Analysis of the MT123 genome for known mechanisms for acid tolerance under mildly and moderately acidic conditions. All four shown systems utilize an amino acid decarboxylase coupled to an antiporter for exchange of the decarboxylase substrate and product. Only the enzymatic steps highlighted in red are predicted in MT123 from the genome analysis. (**A**) Glutamate-dependent pathway. GlsA = glutaminase, GABA = gamma-aminobutyric acid, GadC = glutamate/GABA antiporter, GadA = glutamate decarboxylase A, GadB = glutamate decarboxylase B. (**B**) Arginine-dependent pathway. ArcD = arginine/ornithine antiporter, ArcA = arginine deiminase, ArgF = ornithine carbamoyltransferase, AllK = carbamate kinase, AdiC = arginine/agmatine antiporter, AdiA = arginine decarboxylase. (**C**) Lysine-dependent pathway. CadA = lysine decarboxylase, CadB = cadaverine/lysine antiporter. (**D**) Ornithine-dependent pathway. SpeF = ornithine decarboxylase, PotE = putrescine/ornithine antiporter. Note that the genome has a decarboxylase annotated as an arginine/lysine/ornithine decarboxylase that is a homolog to AdiA, CadA, and SpeF. In all panels: OM = outer membrane, P = periplasm, IM = inner membrane, C = cytoplasm.

In many bacteria, acid tolerance under mildly or moderately acidic conditions depends on the cytoplasmic buffering activity of amino acid decarboxylase enzymes (namely glutamate, arginine, lysine, and ornithine decarboxylases), paired with an amino acid antiporter systems that exchange extracellular amino acids with the decarboxylation products—resulting in a net decrease of cytoplasmic protons (49, 50) (Fig. 2). Analysis of the MT123 genome revealed that the strain lacks the glutamate-dependent system (Fig. 2A) and had only partial versions of the other three systems (Fig. 2B–D). The MT123 genome encodes a putative arginine/lysine/ornithine decarboxylase, which may carry out the decarboxylation of some or all three amino acids. However, the genome lacks the genes encoding the associated antiporters AdiC (arginine/agmatine antiporter), CadB (cadaverine/lysine antiporter), and PotE (putrescine/ornithine antiporter). Another acid tolerance system involves arginine deamination to citrulline (Fig. 2B). This is followed by conversion of citrulline to ornithine and carbamoyl phosphate. Ornithine is exchanged for arginine by an antiporter, and the carbamoyl phosphate is decarboxylated by carbamate kinase to yield ammonium. MT123 encodes the enzyme ArgF (ornithine carbamoyltransferase) for the second step in the pathway; however, it lacks a homolog for the arginine deaminase ArcA, as well as the ArcD antiporter and the carbamate kinase AllK.

At very low pH, acid-tolerant bacteria also utilize the urease system (encoded by the *ureIABEFG* operon) to produce alkali in the form of ammonia (51). However, this system is absent in the MT123 genome. Proteins previously associated with acid tolerance responses (49) that are encoded in the MT123 genome include RecA (repair and maintenance of DNA damage), DnaK (chaperone Hsp70)*,* DnaJ (chaperone Hsp40), ClpXP (protease), ClpAP (protease), GroEL (chaperone Hsp60), GroES (chaperone Hsp10), and UvrABC (excinuclease). We next examined if any of these identified systems were involved in the MT123 growth at mildly acidic pH or if the cells utilized a yet-undescribed system for acid tolerance.

### Proteomic changes during growth at pH 5.5

We compared the proteomic response of cells grown at pH 5.5 to cells grown at pH 7.5 under both aerobic and (anaerobic) denitrifying growth conditions. Differentially abundant proteins at pH 5.5 were identified through comparison to the proteome of cultures grown at pH 7.5 (notated as Log2FC). When considering both the aerobic and denitrifying growth conditions, we detected 353 significantly (adjusted *P* < 0.05) differentially abundant proteins at pH 5.5 (Tables S5 to S8). However, the proteomic response to growth at pH 5.5 was more pronounced under aerobic conditions (310 differentially abundant proteins) compared to denitrifying conditions (43 differentially abundant proteins). Notably, none of the putative acid tolerance proteins identified in our genome analysis showed a significant change in abundance at pH 5.5 under either growth condition. Instead, we observed that both glutamate metabolism and BCAA metabolism were significantly altered in the proteome at pH 5.5 under both growth conditions (Table 1). Thus, we examined the involvement of both sets of metabolic pathways in growth under mildly acidic conditions.

**TABLE 1:** Genes involved in glutamate metabolism or BCAA metabolism with large fitness changes in pH 5.5 compared to pH 7.5^*a*^

Locus ID	Annotation	Aerobic growth		Denitrifying growth	
		*Δfit*_pH 5.5-pH7.5_	Proteome Log2FC	*Δfit*_pH 5.5-pH7.5_	Proteome Log2FC
Glutamate metabolism					
GFF134	Bifunctional glutamine synthetase adenylyltransferase/adenylyl-removing enzyme	+0.31	−0.82	**+1.00**	−0.12
GFF861	Glutamate dehydrogenase	**+1.18**	**−2.56**	**+2.16**	−0.64
GFF1855	Aromatic-amino-acid aminotransferase	+0.15	+0.13	**−3.53**	+0.06
GFF2287	Anthranilate synthase component 1	−0.06	+0.76	**−2.58**	+0.30
GFF2291	Anthranilate synthase component 2	+0.16	+0.80	**−2.11**	+0.58
GFF2390	Glutamate 5-kinase	**+2.64**	+0.54	+0.44	+0.23
GFF2471	2-aminoadipate transaminase	+0.07	+0.02	**−3.68**	−0.12
GFF2936	Glutamate synthase small chain	**−2.91**	**+3.32**	**−3.54**	+0.93
GFF2937	Glutamate synthase large chain	**−3.10**	**+3.05**	**−3.78**	**+1.01**
GFF3010	Glutamate-pyruvate aminotransferase AlaC	**+1.17**	−0.04	−0.31	−0.09
GFF664	Glutarate-semialdehyde dehydrogenase DavD	+0.18	**+4.86**	+0.17	+3.18
BCAA metabolism					
GFF233	High-affinity branched-chain amino acid transport ATP-binding protein LivF	−0.01	**−2.44**	+0.20	−0.09
GFF370	Leucine-, isoleucine-, valine-, threonine-, and alanine-binding protein	0.00	**−5.84**	+0.02	−0.14
GFF590	Leu/Ile/Val-binding protein	0.00	**−2.15**	−0.01	**−1.64**
GFF662	Leu/Ile/Val-binding protein	+0.21	**+2.53**	+0.11	+1.34
GFF824	Leu/Ile/Val-binding protein	+0.03	**−10.14**	−0.05	+0.21
GFF860	L-threonine 3-dehydrogenase	+0.07	**−1.29**	+0.39	+0.22
GFF866	Leucine-, isoleucine-, valine-, threonine-, and alanine-binding protein	+0.07	**−7.21**	−0.17	−0.51
GFF1331	Leu/Ile/Val-binding protein	+0.05	**−9.74**	+0.09	+0.72
GFF1401	Leu/Ile/Val-binding protein	+0.01	**−10.23**	+0.01	+0.72
GFF1455	Acetolactate synthase isozyme 3 large subunit	**+2.17**	+0.77	−0.49	+0.18
GFF1489	Leucine-, isoleucine-, valine-, threonine-, and alanine-binding protein	+0.01	**−10.01**	+0.1	−2.71
GFF1694	3-isopropylmalate dehydratase small subunit 1	**+2.13**	+0.23	**+1.22**	−0.27
GFF1695	3-isopropylmalate dehydratase large subunit 1	**+2.16**	+0.05	−0.11	−0.23
GFF1883	Leu/Ile/Val-binding protein	−0.03	**−2.52**	−0.09	−1.17
GFF1887	High-affinity branched-chain amino acid transport ATP-binding protein LivF	−0.08	**−2.17**	+0.14	−0.09
GFF1948	Leucine-, isoleucine-, valine-, threonine-, and alanine-binding protein	+0.02	**−4.52**	−0.89	−4.08
GFF1966	High-affinity branched-chain amino acid transport ATP-binding protein LivF	**+1.07**	**−2.22**	**+2.43**	−0.61
GFF1969	High-affinity branched-chain amino acid transport system permease protein LivH	**+1.25**	nd	**+2.38**	nd
GFF1970	Leucine-, isoleucine-, valine-, threonine-, and alanine-binding protein	**+1.18**	**−4.76**	**+2.22**	−2.13
GFF1976	Leucine-, isoleucine-, valine-, threonine-, and alanine-binding protein	+0.47	**−2.88**	+0.90	−1.28
GFF2011	Leu/Ile/Val-binding protein	+0.11	**−9.35**	−0.05	+0.63
GFF2121	High-affinity branched-chain amino acid transport ATP-binding protein LivF	−0.05	**−2.57**	−0.38	−2.79
GFF2125	Leu/Ile/Val-binding protein	+0.14	**−3.74**	−0.28	−4.26
GFF2575	Leucine-, isoleucine-, valine-, threonine-, and alanine-binding protein	−0.01	**−7.25**	+0.03	+0.19
GFF2717	2-isopropylmalate synthase	**+2.71**	0.27	−0.24	−0.16
GFF2848	Leucine-, isoleucine-, valine-, threonine-, and alanine-binding protein	+0.02	**−6.16**	+0.13	−1.28
GFF2852	High-affinity branched-chain amino acid transport ATP-binding protein LivF	−0.02	**−8.22**	**−1.18**	−1.80

^*a*^ Δfit_pH5.5-pH7.5_ values represent gene fitness value changes between growth at pH 5.5 and pH 7.5, calculated from RB-TnSeq data. Significant fitness changes are bolded and defined as |fitness change| ≥ 1. Accompanying Log2FC values represent fold changes from proteomics data, with significant fold changes in bold and defined as |Log2FC| ≥ 1 with an adjusted *P*-value < 0.05. nd, protein not detected. The linked RefSeq locus tags can be found in Table S2.

### Proteomic and metabolomic analyses suggest production of glutamate during pH 5.5 growth as an acid tolerance mechanism

**Fig 3:**
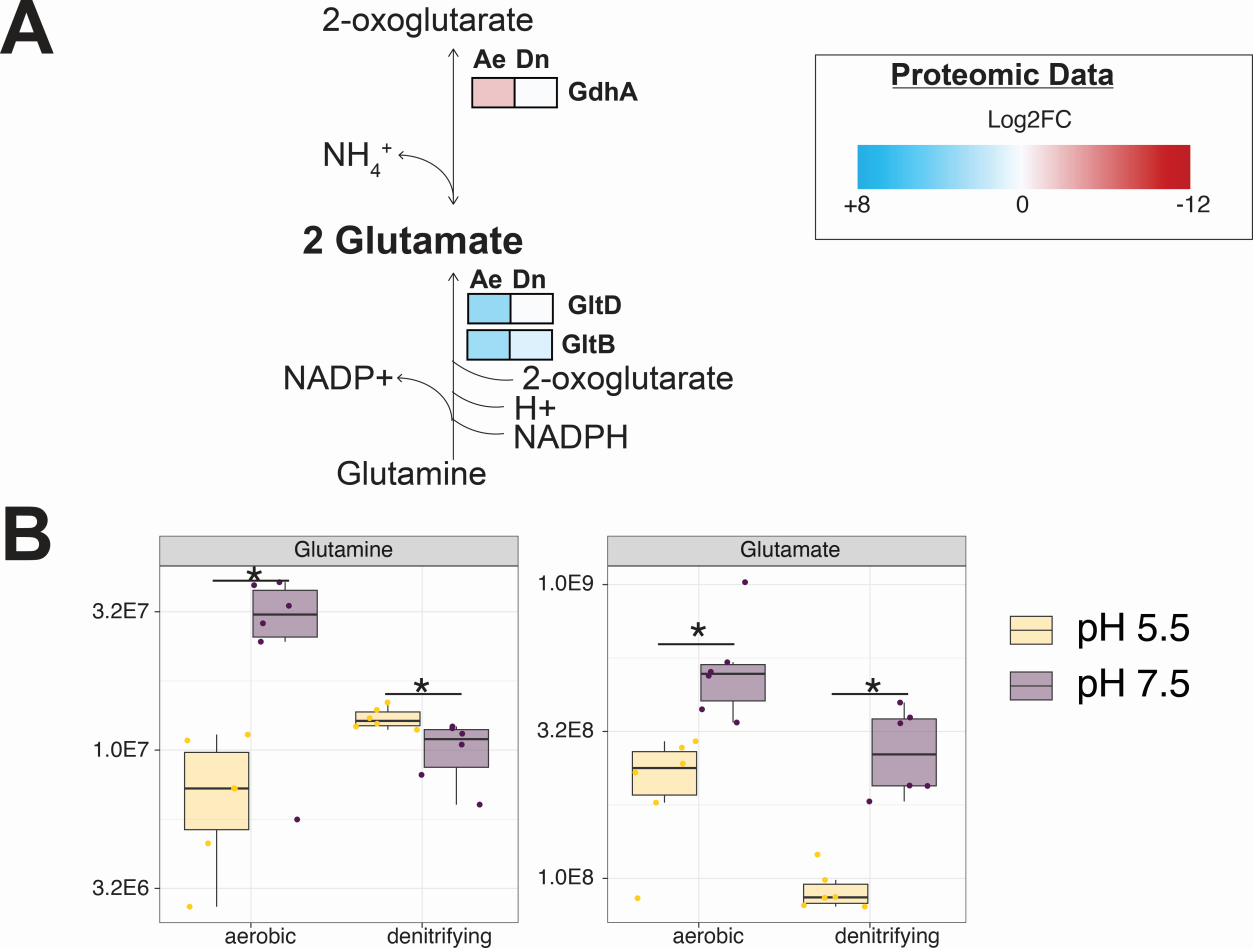
Integrated proteomic and metabolomic insights into the role of glutamate metabolism in the cellular response to growth under mildly acidic conditions. (**A**) Patterns of protein abundances involved in glutamate metabolism. Heat maps display the average (*n* = 3) Log2FC values for the individual proteins under aerobic (Ae) and denitrifying (Dn) growth conditions. The heat map scale (displayed as Log2FC abundances at pH 5.5 vs 7.5) is at the bottom of the panel. Blue boxes indicate increased protein abundance (adjusted *P* < 0.05), red boxes indicate proteins that were significantly decreased in abundance (adjusted *P* < 0.05), and white boxes indicate no significant difference in protein abundance. Statistical comparisons were performed with a Welch’s *t*-test using the Benjamini-Hochberg procedure to control for the false discovery rate (FDR). (**B**) Relative abundances of intracellular glutamine and glutamate. Individual data points represent replicate experiments (*n* = 6). Statistical comparisons were performed with a Welch’s *t*-test using the Benjamini-Hochberg procedure to control for the false discovery rate (FDR). Asterisks (*) indicate adjusted *P*-values < 0.05.

**Fig 4:**
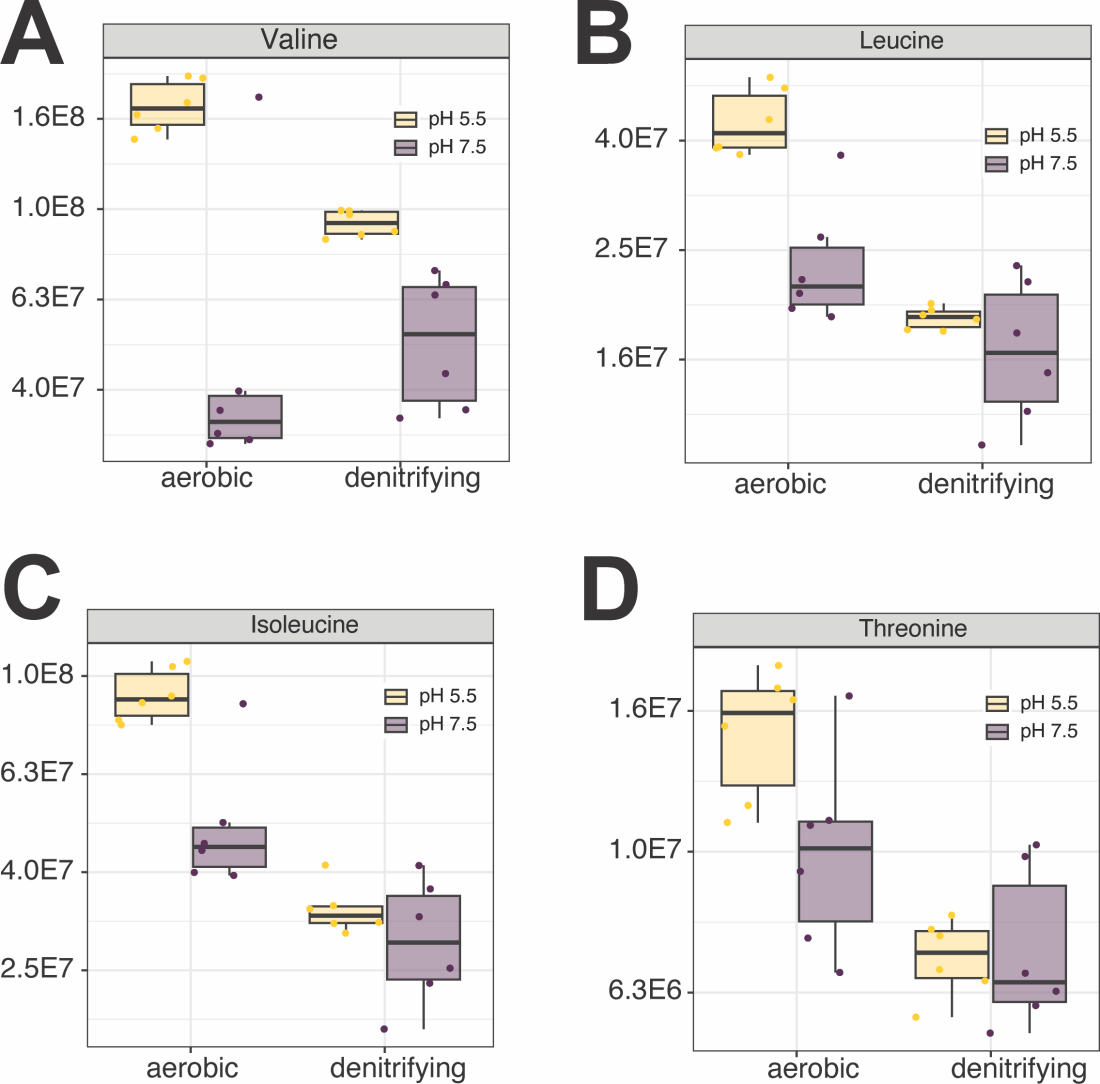
Changes in intracellular abundances of BCAAs and threonine during growth under mildly acidic conditions. Intracellular abundances of valine (**A**), leucine (**B**), isoleucine (**C**), and threonine (**D**). For all panels, individual data points represent replicate experiments (*n* = 6). Statistical comparisons were performed with a Welch’s *t*-test using the Benjamini-Hochberg procedure to control for the false discovery rate (FDR). Asterisks (*) indicate adjusted *P*-values < 0.05.

As a general trend at pH 5.5, we observed an increase in the abundance of enzymes that catalyze glutamate-yielding reactions and a decrease in the abundance of enzymes that catalyze glutamate-consuming reactions (Table 1). For example, we observed an increased abundance of the glutamate synthase large subunit (GltB) under both growth conditions (aerobic: +3.0 log2FC, denitrifying: +1.0 Log2FC) and the glutamate synthase small subunit (GltD) under aerobic growth (+3.3 log2FC), both of which are predicted to produce glutamate from glutamine and 2-oxoglutarate (alpha-ketoglutarate) (Fig. 3A) (52). Consistent with this observation, targeted metabolomic analyses revealed significantly decreased (−2.0 Log2FC) cellular glutamine content at pH 5.5 relative to pH 7.5 under aerobic conditions (Fig. 4B; Table S9). In contrast, the glutamate dehydrogenase (GdhA)—which is predicted to consume glutamate, producing ammonium and 2-oxoglutarate (53)—decreases in abundance under aerobic growth conditions (−2.6 log2FC).

At pH 5.5, intercellular glutamate decreased relative to pH 7.5 under both growth conditions (Fig. 3B; Table S9). This is despite the increased abundance of proteins involved in glutamate production and decreased abundance of proteins involved in glutamate consumption. Thus, growth at pH 5.5 appears to increase the cellular demand for glutamate, likely triggered by glutamate depletion. While both the metabolomic and proteomic data were collected at the same points in MT123 growth, the two data types inherently represent “snapshots in time” of different scales. The turnover of metabolites can occur at rates orders of magnitude faster than the turnover of proteins (54–56). The simultaneous increase in the abundances of proteins involved in glutamate production, alongside the decrease in cellular glutamate abundance, suggests consistent and ongoing glutamate consumption occurring at high rates within the cell during growth at pH 5.5.

### BCAA accumulation leads to repression of BCAA transport during pH 5.5 growth

Among the proteins that decreased in abundance during growth at pH 5.5, perhaps the most striking trend was the large number of BCAA transporters. When analyzing the MT123 genome, we observed a remarkably high number of BCAA transporters, including 25 LivH/LivM homologs (the permease subunit). In *E. coli* K12, the Liv transporter is a common transporter for L-leucine, L-isoleucine, L-valine, and the BCAA precursor L-threonine, although the specificity of this transporter may vary among species (57–60). A multiplicity of BCAA transport systems has been reported in other bacteria, likely due to the critical functions of BCAAs as key metabolic intermediates and in signaling of cellular metabolic status (58, 60, 61). However, we failed to find a report of a bacterial genome with as many BCAA transporter homologs as MT123. In the pH 5.5 proteomes, we observed decreases in abundance of five homologs of the high-affinity BCAA transporter ATP-binding subunit LivF and 15 homologs of the BCAA binding protein of the Liv transporter (Table 1), with a greater number of the transporters decreasing in abundance under aerobic growth conditions (20 proteins) compared to denitrifying conditions (one protein).

We next asked whether BCAAs accumulate in the cytoplasm during growth at pH 5.5—a possible signal to decrease the expression of BCAA importers. We observed significant increases in the abundance of valine at pH 5.5 under both aerobic and denitrifying conditions (Fig. 4A; Table S9). We also observed significant increases in leucine and isoleucine abundances at pH 5.5 under aerobic growth conditions (Fig. 4B and C; Table S9). While leucine and isoleucine abundances did increase at pH 5.5 under denitrifying conditions, this increase did not achieve the threshold of significance (Fig. 4B and C; Table S9). No significant changes in abundances of the BCAA-precursor threonine were observed under either condition (Fig. 4D; Table S9). Thus, we suggest that under mildly acidic conditions, intracellular concentrations of the BCAAs leucine, valine, and isoleucine increase, leading to repression of BCAA importers. In other gram-negative bacteria, leucine, valine, threonine, and isoleucine are effectors of the global regulator Lrp (***L***eucine-responsive ***r***egulatory ***p***rotein) (62, 63), with leucine-bound Lrp acting as a repressor of the *liv* operon in *E. coli* K12 (64). MT123 encodes three Lrp homologs in its genome. We propose that the accumulation of BCAAs during growth under mildly acidic conditions leads to Lrp-mediated repression of its many *liv* operons.

### Opposing effects of glutamate and BCAAs on MT123 fitness during mild acid stress

While the proteomic and metabolomic data reveal the cellular response to mildly acidic conditions, these data do not imply whether these responses benefit the cells under these conditions. Thus, we constructed an RB-TnSeq pooled mutant library to calculate fitness scores for 2,349 genes during growth at pH 5.5 (approximately 75% of MT123’s predicted protein coding genes). Changes in gene fitness values (Δ*fitness*) were calculated from the difference between gene fitness values of pH 5.5 and pH 7.5 cultures (Table S10). Genes with large positive (Δ*fitness* > 1) or large negative (Δ*fitness* < −1) fitness changes (35) were analyzed further for involvement in either glutamate or BCAA metabolism. Negative fitness changes reflect gene disruptions that have a more detrimental effect on growth at pH 5.5 relative to pH 7.5. Positive fitness changes reflect gene disruptions that have a more beneficial effect on growth at pH 5.5 relative to pH 7.5.

**Fig 5:**
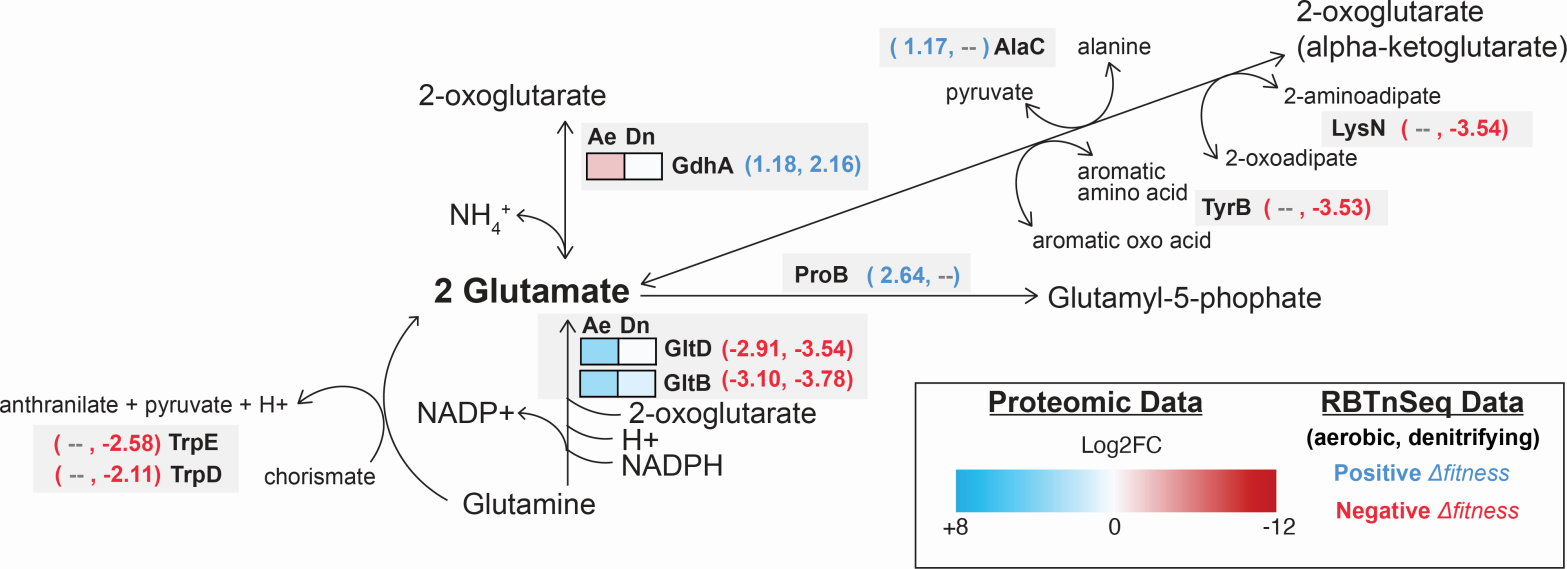
Integrated proteomic and mutant fitness assay (RB-TnSeq) insights into the role of glutamate metabolism in the cellular response to growth under mildly acidic conditions. Heat maps display the average (*n* = 3) Log2FC values for the individual proteins under aerobic (Ae) and denitrifying (Dn) growth conditions. The heat map scale (displayed as Log2FC abundances at pH 5.5 vs 7.5) is at the bottom of the panel. Blue boxes indicate increased protein abundance (*P* < 0.05), red boxes indicate proteins that were significantly decreased in abundance (*P* < 0.05), and white boxes indicate no significant difference in protein abundance. Statistical comparisons were performed with a Welch’s *t*-test using the Benjamini-Hochberg procedure to control for the false discovery rate. These data are overlaid with mutant fitness data for the gene encoding the enzyme. Genes with large positive (Δfitness > 1) or large negative (Δfitness < −1) are highlighted by the bracketed numbers next to the heat maps in either blue (positive) or red (negative) text. The first number is the Δfitness under aerobic conditions, and the second is under denitrifying conditions. If an enzyme lacks accompanying mutant fitness data, it means that there was no large Δfitness under either growth condition.

The RB-TnSeq data support a model where glutamate accumulation is critical in the cellular response to growth at pH 5.5 (Table 1). Highly negative Δ*fitness* values were observed for the glutamate synthase genes (*gltDB*) under both aerobic and denitrifying growth conditions (*Δfitness_aerobic_ =* −2.91 and −3.10; *Δfitness_denitrifying_ =* −3.54 and −3.78), consistent with our observation that these proteins increase in abundance at pH 5.5 (Fig. 5). The screen also uncovered additional genes predicted to be involved in glutamate production with negative Δ*fitness* values, including those encoding the 2-aminoadipate transaminase (*lysN*) (65), the aromatic amino acid aminotransferase (*tyrB*) (66), and the anthranilate synthase subunits 1 (*trpE*) and 2 (*trpD*) (67). As amino acid transaminases are readily reversible (68, 69), we suggest that under the growth conditions utilized here, they might operate in the direction of glutamate synthesis (Fig. 5). In contrast, we observed that genes encoding enzymes predicted to be involved in glutamate consumption had positive Δ*fitness* values, suggesting that glutamate consumption through these pathways is detrimental to cell fitness at pH 5.5 (Table 1). For example, the glutamate dehydrogenase *(gdhA)* gene had positive Δ*fitness* values (*Δfitness_aerobic_ = +*1.18; *Δfitness_denitrifying_ = +*2.16). Consistent with this observation, the glutamate dehydrogenase (GdhA) decreased in abundance at pH 5.5 under aerobic growth conditions (Fig. 5). We also found that the glutamate 5-kinase gene (*proB*), which converts glutamate to glutamyl-5-phosphate (70), and the glutamine synthetase adenylyltransferase (glpE), which regulates the activity of the glutamine synthetase (GlnA; glutamate → glutamine) (71), had positive Δ*fitness* values under aerobic growth conditions (Table 1). In contrast to the other two transaminase-encoding genes, the *alaC* gene—encoding the glutamate-pyruvate aminotransferase (72)—had a positive Δ*fitness* under aerobic conditions, suggesting that it may be active primarily in the direction of 2-oxoglutarate and alanine formation from glutamate and pyruvate (Fig. 5).

**Fig 6:**
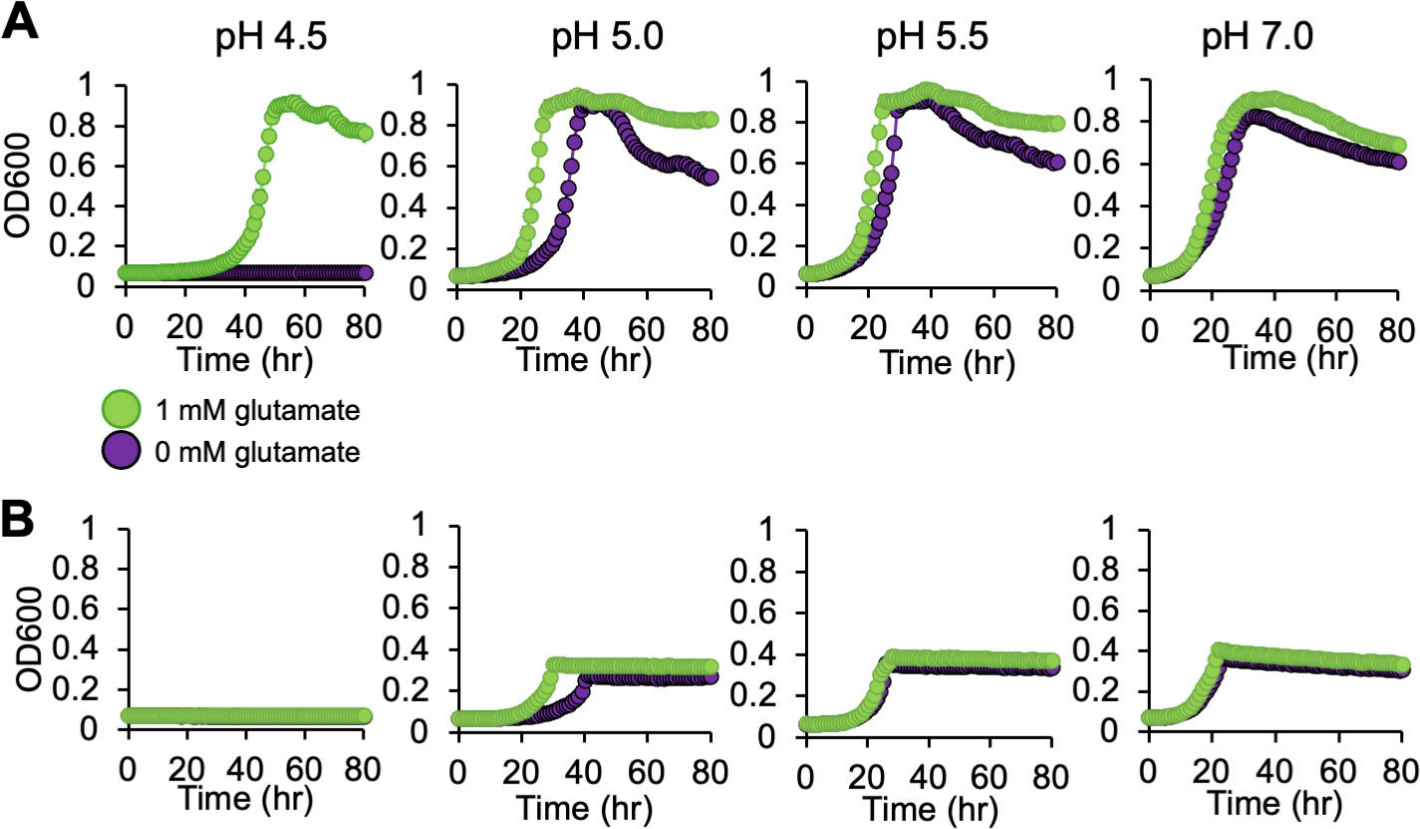
Growth of MT123 at varying pHs with 1 mM glutamate. Growth curves were performed under aerobic (**A**) and denitrifying (**B**) growth conditions. Individual points represent the average of three replicates. Error bars are present, though not always visible, and represent ±SD. The on-figure legend applies to all panels.

These proteomic, metabolomic, and mutant fitness screen data suggest that intracellular glutamate is key for cellular growth at pH 5.5. Therefore, we next tested the impact of exogenous glutamate on growth under acidic conditions. We grew MT123 under both aerobic (Fig. 6A) and denitrifying (Fig. 6B) growth conditions at increasingly lower pHs until growth was eliminated (pH 4.5 for both conditions). We then grew the cells at pH 4.5, 5.0, 5.5, and 7.0 with the addition of 1 mM glutamate. Glutamate rescued any growth defects at pH 4.5 and 5.0 under aerobic conditions and at pH 5.0 under denitrifying conditions. Above pH 5.0, the growth improvement from the glutamate was minimal since growth was not inhibited at those pHs. While the MT123 genome does not encode a GadC homolog (Fig. 2A), MT123 does seem to have the ability to import glutamate. We searched the MT123 genome for homologs of the *E. coli* GltS, GltP, and GltIJKL glutamate transporters. Putative homologs were identified for GltP (GFF2148) and GltIJKL (GFF926-929). Both GltP and GltIJKL have been linked to acid tolerance in other bacteria (73, 74). Oddly, we observed decreases in the abundance of GltI (aerobic: −2.7 Log2FC, denitrifying: −1.5 Log2FC) and GltJ (aerobic: −1.0 Log2FC, denitrifying: −1.0 Log2FC) at pH 5.5 in our proteomics data. A second GltI homolog (GFF1522) was increased in abundance at pH 5.5 under aerobic conditions (+1.9 Log2FC). However, the proteomics experiments were performed without glutamate supplementation, where *de novo* synthesis is likely the major source of cytoplasmic glutamate pools rather than uptake—making it challenging to interpret these data in the context of our glutamate supplementation experiments.

Given the negative Δ*fitness* observed for *gltDB*, we considered that the glutamate synthase-catalyzed conversion of glutamine to glutamate might be important for low pH tolerance due to the consumption of a cytoplasmic proton in the process (Fig. 5). Thus, we performed these same experiments with 1 mM glutamine. However, glutamine did not rescue growth at pH 4.5 and only slightly decreased lag time at pH 5.0 (Fig. S1). Taken together, our results suggest that the accumulation of glutamate itself is critical for MT123 growth under mildly acidic conditions. We propose that the modulation of enzyme abundances (Table 1) to increase metabolite flux toward glutamate (and decrease its consumption via certain pathways) is an acclimatization response to mildly acidic conditions during both aerobic and denitrifying growth.

In contrast to glutamate, our mutant fitness data support a model in which the accumulation of one or more of the BCAAs is detrimental to cellular growth at pH 5.5. While there is likely some degree of functional redundancy among the multiple BCAA transporters (58) in the MT123 genome, a cluster of genes encoding three of the Liv subunits (GFF1966, 1969, 1970) all had positive Δ*fitness* values under aerobic and denitrifying growth conditions (Table 1). This Liv transporter also decreased in abundance at pH 5.5 under aerobic growth conditions (Table 1). Consistent with our model, we also observed positive Δ*fitness* values for several genes involved in BCAA biosynthesis. This included the gene encoding the acetolactate synthase large subunit (*ilvI, Δfitness_aerobic_ =* +2.17), which is part of both the isoleucine and valine biosynthesis pathways. Additionally, we observed Δ*fitness* values for the 3-isopropylmalate dehydratase large (*leuC, Δfitness_aerobic_ =* +2.13; *Δfitness_denitrifying_ =* +1.22) and small subunit (*leuD, Δfitness_aerobic_ =* +2.16), as well as the 2-isopropylmalate synthase (*leuA, Δfitness_aerobic_ =* +2.71)—all part of the leucine biosynthesis pathway.

**Fig 7:**
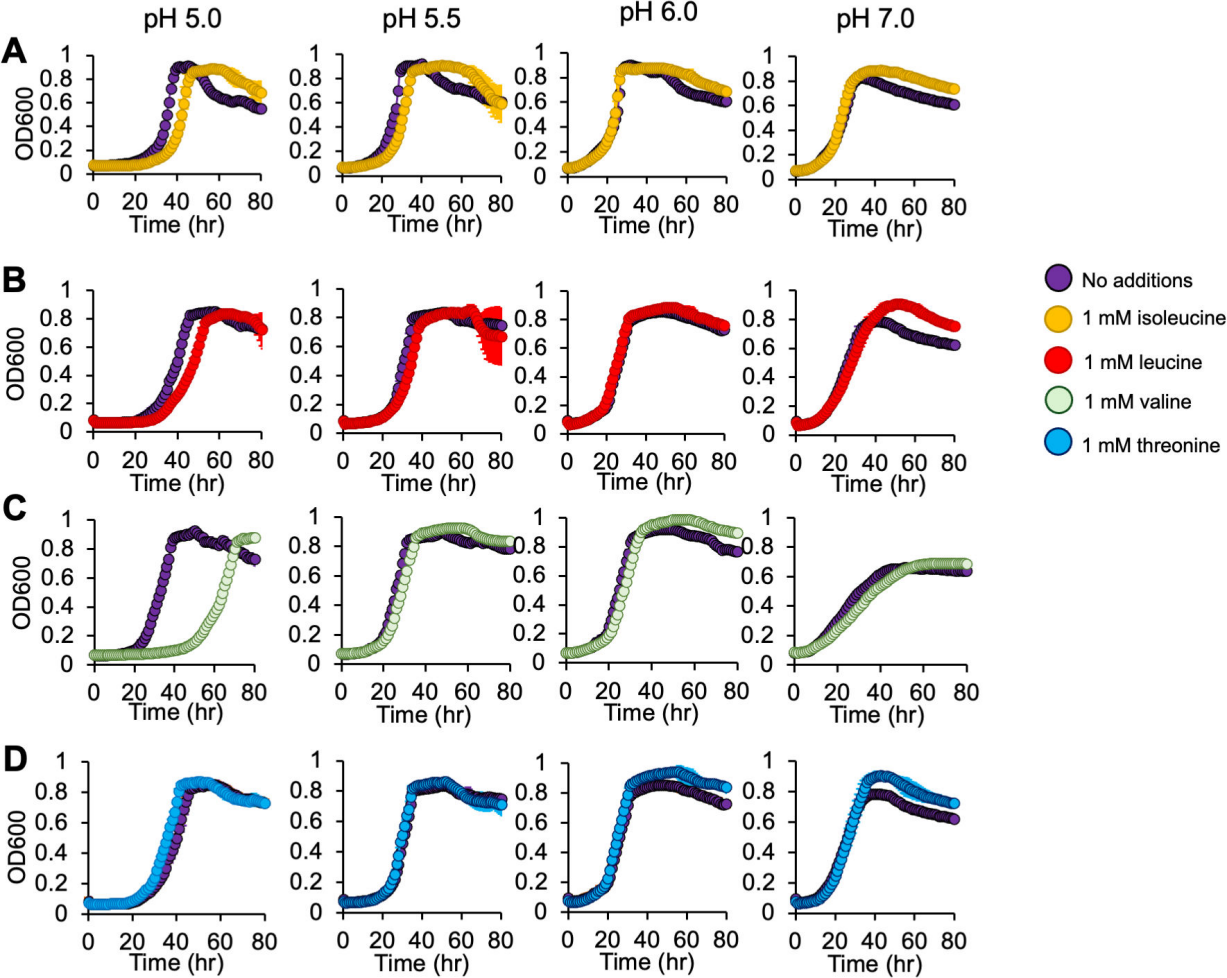
Growth of MT123 at varying pHs with 1 mM BCAA and threonine additions. Growth curves were performed under aerobic growth conditions. (**A**) Growth with 1 mM isoleucine. (**B**) Growth with 1 mM leucine. (**C**) Growth with 1 mM valine. (**D**) Growth with 1 mM threonine. Individual points represent the average of three replicates. Error bars are present, though not always visible, and represent ±SD. The on-figure legend applies to all panels.

**Fig 8:**
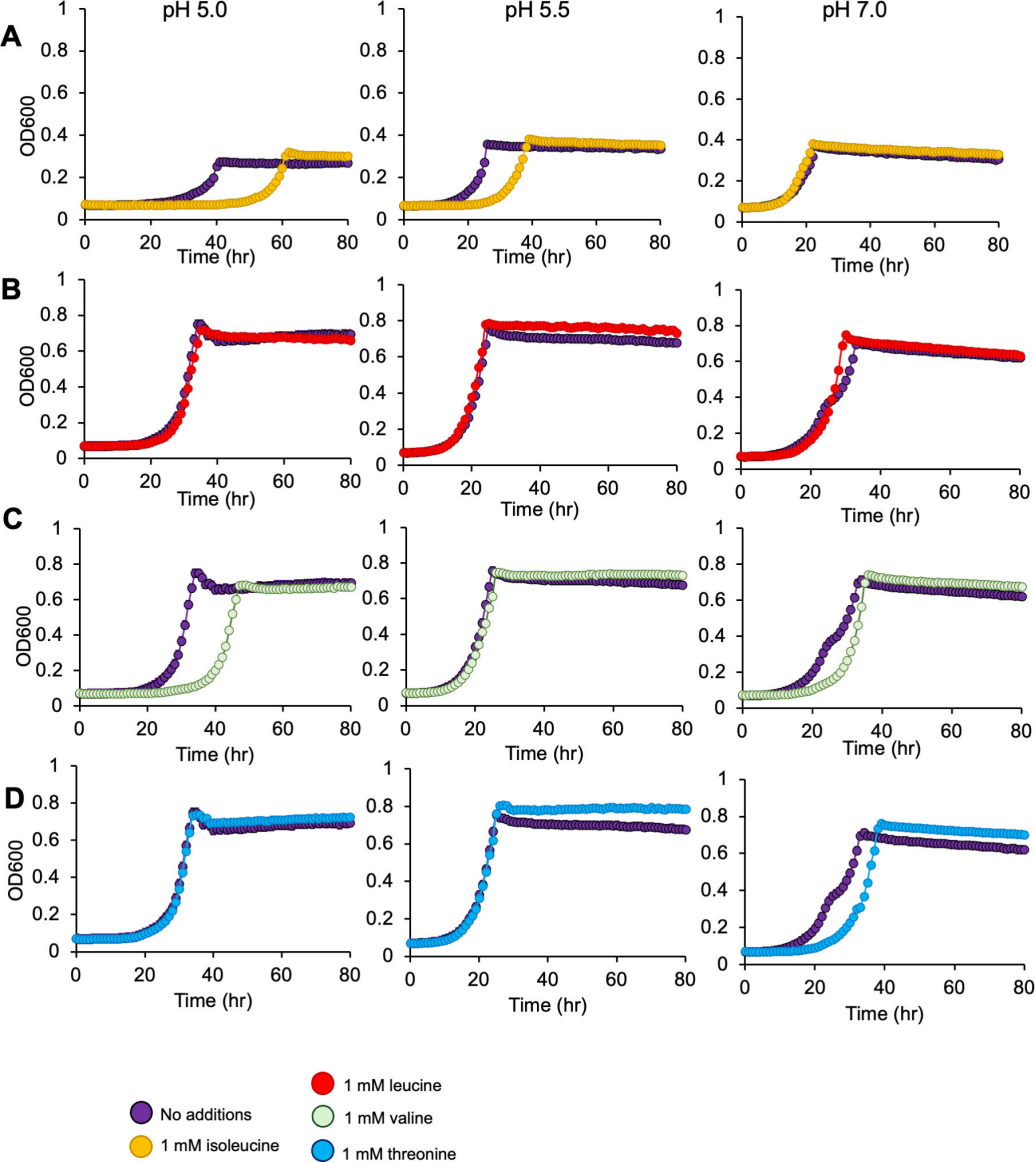
Growth of MT123 at varying pHs with 1 mM BCAA and threonine additions. Growth curves were performed under denitrifying growth conditions. (**A**) Growth with 1 mM isoleucine. (**B**) Growth with 1 mM leucine. (**C**) Growth with 1 mM valine. (**D**) Growth with 1 mM threonine. Individual points represent the average of three replicates. Error bars are present, though not always visible, and represent ±SD. The on-figure legend applies to all panels.

From these data, we hypothesized that accumulation of leucine, isoleucine, and/or valine—as was observed in our pH 5.5 metabolomics data (Fig. 4)—is inhibitory to cell growth at pH 5.5. We tested this hypothesis by repeating the same series of amino acid addition growth experiments performed above, but with 1 mM additions of isoleucine, leucine, valine, and threonine under both aerobic (Fig. 7) and denitrifying growth conditions (Fig. 8). Additions of isoleucine, leucine, or valine all inhibited growth at pH 5.0, with valine as the most inhibitory. These effects were more pronounced under denitrifying compared to aerobic growth conditions. In contrast, amendments with the BCAA precursor threonine had no impact on growth under the conditions tested. Considering these findings, we suggest that the Liv transporter (GFF1966, 1969, 1970) (Table 1), with positive Δ*fitness* values, is likely the primary transporter for leucine, isoleucine, and/or valine import by MT123 under the conditions studied here.

### Summary of findings

The proteomic, metabolomic, and RBTn-Seq data presented above provide distinct yet complementary views of the cellular state during low pH stress. The proteomic and metabolomic data sets represent different temporal scales of the cellular response to the stressor: metabolite turnover occurs at orders of magnitude faster rates than protein turnover (54–56). However, both reflect the cellular response to the stressor. Importantly, neither data set alone indicates whether these responses improve or decrease cellular fitness at pH 5.5. RB-TnSeq clarifies this latter point using high-throughput phenotyping of mutant strains: positive *Δfitness* values show that a gene is detrimental to growth at pH 5.5, whereas negative values indicate the opposite. Across these three data types, glutamate and BCAA metabolism produced distinctly opposing effects. At pH 5.5, proteins involved in glutamate biosynthesis increased in abundance, while those involved in its consumption decreased—consistent with the negative *Δfitness* values for glutamate-biosynthetic genes (Table 1; Fig. 5). Yet, intracellular glutamate remained low (Fig. 3B), suggestive of substantial downstream use. Together, these results suggest that glutamate production is important for acid tolerance, possibly due to downstream demands for this amino acid (discussed below). In contrast, at pH 5.5, the abundance of proteins involved in BCAA biosynthesis and transport decreased (Table 1). The positive *Δfitness* values for many of these genes suggest that intracellular BCAA accumulation may be detrimental under acidic conditions (Table 1). Consistent with this, we observed elevated intracellular BCAA levels at pH 5.5 (Fig. 4), suggesting that such accumulation may be one mechanism by which low pH inhibits growth (discussed below).

## DISCUSSION

While glutamate is often implicated in the bacterial response to mildly acidic growth conditions, MT123 lacks the canonical glutamate-based acid tolerance system consisting of the glutaminase GlsA, glutamate decarboxylases GadA and GadB, and the glutamate/gamma-aminobutyric acid (GABA) antiporter GadC (49) (Fig. 2A). Even so, our data indicate that at pH 5.5, MT123 redirects metabolic flux toward glutamate, and that this increased intracellular glutamate pool (whether endogenously generated or supplemented externally) is important for fitness under acid stress. Because glutamate can be further decarboxylated to GABA—coupled to proton consumption—in many bacteria grown under acidic conditions (Fig. 2A), we considered whether MT123 might be channeling glutamate toward GABA as part of its acid tolerance response. Our metabolomic profiles indeed show that intracellular GABA increases significantly at pH 5.5 relative to 7.5 (Fig. S2). The two pathways that could potentially account for GABA formation in MT123 are: (i) decarboxylation of glutamate or (ii) putrescine degradation. The putrescine route appears unlikely because our medium lacks putrescine as a substrate. Additionally, PuuE, the reversible aminotransferase involved in this pathway, does not change in abundance at pH 5.5. Together, these data support the interpretation that MT123 most likely produces GABA via decarboxylation of glutamate through an uncharacterized decarboxylase, despite lacking the canonical GadA/GadB system.

The proteomic and mutant fitness data provide further insight into this speculative glutamate decarboxylase system, suggesting potential targets for future study. In our proteomic data, an enzyme annotated as an aspartate 1-decarboxylase (PanD) (75) is increased in abundance at pH 5.5 (relative to 7.5) during aerobic growth. This protein was also detected under denitrifying conditions; however, its Log2FC did not reach our threshold of significance. We suggest that this decarboxylase might have a broader substrate specificity, allowing it to decarboxylate either glutamate or aspartate. Substrate promiscuity has been reported previously in amino acid decarboxylases (76, 77). However, there were no significant mutant fitness effects associated with the gene (*panD*) encoding this enzyme. Thus, MT123 might have multiple enzymes with redundant glutamate decarboxylase activities. For example, there are two additional amino acid decarboxylases encoded in the MT123 genome: arginine/ornithine decarboxylase (AdiA/CadA/SpeF homolog shown in Fig. 2) and a glycine decarboxylase (GcvP) (Table S1). In addition, there are other non-amino acid decarboxylases annotated in the MT123 genome, including a homoprotocatechuate decarboxylase, phosphatidylserine decarboxylase, oxaloacetate decarboxylase, benzoylformate decarboxylase, orotidine 5’-phosphate decarboxylase, diaminopimelate decarboxylase, and uroporphyrinogen decarboxylase (Table S1).

**Fig 9:**
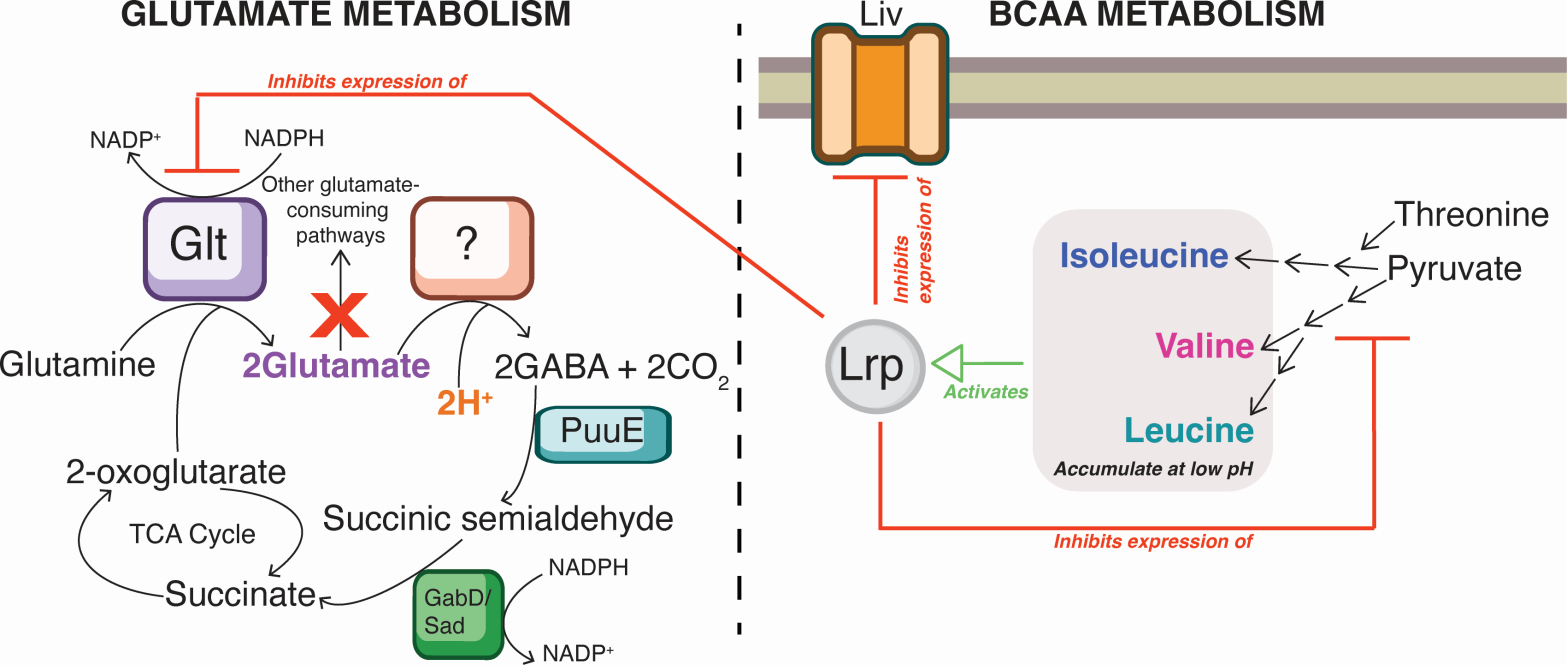
Conceptual model for the MT123 response to acidic growth conditions. Glutamate synthesis and decarboxylation to form GABA are shown in the left panel. The GABA is then recycled back to 2-oxoglutarate through the GABA shunt and the TCA cycle. In the right panel, BCAAs are seen to accumulate under acidic conditions. These BCAAs activate the global regulator Lrp, which can then repress the expression of BCAA biosynthetic genes, the Liv BCAA transporter, and the Glt glutamate synthase.

We propose that MT123 maintains cytoplasmic pH under mildly acidic conditions through a glutamate-dependent mechanism in which glutamate decarboxylation consumes a cytoplasmic proton, likely via an unidentified decarboxylase (discussed above) (78) (Fig. 9). The glutamate substrate for this reaction may be generated from glutamine via the glutamate synthase. Although the glutaminase typically carries out this reaction in other bacteria, this enzyme is absent from the MT123 genome (Fig. 2A). However, several reports have also noted that glutamate synthase can fulfill this role in other bacteria (79, 80). The glutamate synthase also directly consumes protons during glutamine conversion to glutamate; however, the relevance of this to overall acid tolerance in MT123 remains ambiguous since glutamine supplementation only mildly improved growth at the lower pH.

A remaining question is the identity of the antiporter system that is needed to exchange GABA for exogenous glutamate following the decarboxylation reaction (81). However, prior work has suggested that endogenous glutamate may contribute partially, or fully, to acid tolerance in certain bacteria (82). For example, *Mycobacterium* species have glutamate-based acid tolerance systems, yet lack a GadC homolog (83). Decoupled intracellular GABA synthesis and efflux in response to low pH has also been reported in *Listeria monocytogenes* (84). In these organisms, it is speculated that GABA is recycled through the GABA shunt to succinate and then back to 2-oxoglutarate, involving the activities of a GABA aminotransferase and the succinate-semialdehyde dehydrogenase GabD/Sad. The MT123 genome encodes an intact GABA shunt (82) (Fig. 9). Interestingly, the GabD/Sad homolog (GFF664) increased in abundance at pH 5.5 under aerobic growth conditions (+4.9 log2FC) (Table 1). However, the GABA aminotransferase of MT123, PuuE, did not change in abundance under either growth condition, although it was still detectable. We note that some bacteria, such as *Rhizobium leguminosarum*, have multiple transaminases with GABA transaminase activity (85). However, more work is required to fully characterize these non-canonical acid tolerance systems in MT123 and other bacteria.

In contrast to glutamate, which enhances low-pH tolerance, the BCAAs display nearly the opposite physiological effect—one that is also more challenging to decipher, since this class of amino acids has not been previously linked to acid stress. Additionally, the findings related to the BCAAs should be interpreted cautiously as their cytoplasmic accumulation can produce broad, pleiotropic effects on cellular metabolism. For example, valine induces isoleucine starvation in many bacterial species via feedback inhibition on the acetohydroxamic synthase (86–88), which triggers a broad starvation response. Leucine is also an effector of the global regulator Lrp, encoded by three homologous genes in the MT123 genome (64). However, this regulatory complexity also raises the interesting question of whether BCAA metabolism intersects with glutamate metabolism during acid stress. One direct link is that glutamate donates an amino group to the keto-acid precursors of isoleucine, leucine, and valine (89, 90). Thus, BCAA synthesis consumes glutamate—a metabolite critical for MT123’s acid acclimation. While this could explain the positive Δ*fitness* values for the BCAA biosynthetic genes (Table 1), it does not account for the low-pH-specific toxicity of exogenous BCAAs (Fig. 7), nor the positive Δ*fitness* effects observed for the genes encoding the BCAA transporter and decreased transporter protein abundance at pH 5.5 (Table 1). Instead, our metabolomic data show that BCAAs accumulate intracellularly under acidic conditions—possibly due to a yet-unknown stress-induced dysregulation of BCAA biosynthesis (91–93) (Fig. 9). Leucine (and likely also isoleucine and valine) (62, 63, 94) modulates the global regulator Lrp, which regulates amino acid biosynthesis, uptake, and degradation, as well as other metabolic functions (95) (Fig. 9). Interestingly, with respect to leucine, the *E. coli* Lrp is a repressible activator of the glutamate synthase gene (*glt*) (96) (Fig. 9). Thus, we hypothesize that the decreases in the abundances of Liv transporters and proteins involved in BCAA biosynthesis during growth at pH 5.5 may minimize this feedback inhibition on the glutamate biosynthesis required to sustain growth under acidic conditions. However, the complexity of the Lrp regulon means that further work is required to disentangle these interactions.

Finally, we made an interesting observation that there were more differentially abundant proteins under aerobic conditions (310 proteins) compared to denitrifying conditions (43 proteins). This is despite the fact that we observed—both here and previously (24)—that MT123 has greater pH tolerance under aerobic conditions compared to denitrifying conditions. Protein synthesis (97) and turnover (98–100) are both ATP-dependent processes. However, under denitrifying conditions, growth at lower pH leads to accumulation of the protonophore nitrous acid (the acidic, protonated form of nitrite—a denitrification intermediate), collapsing the cellular proton motive force (48). Prior studies have found that, in low quantities, nitrous acid can inhibit other ATP-dependent processes (47, 101). During anaerobic growth with nitrate, transient accumulation of nitrite is observed in MT123 cultures in the range of 200 to 300 μM (Table S11), which should be sufficient to yield inhibitory amounts of FNA (47, 102). Indeed, our proteomic data also suggest the accumulation of FNA: under anaerobic conditions at pH 5.5, the NirK nitrite reductase is more abundant than at pH 7.5 (Table S7). We observed a similar increase under aerobic conditions as well (Table S5), suggesting that elevated NirK expression is part of MT123’s general response to acidic environmental conditions, where nitrogen oxides are also common. Thus, we propose that the accumulation of FNA during denitrifying growth at pH 5.5 might contribute both to the reduced proteomic response and to the greater growth defects observed under denitrifying conditions.

In summary, we show how a multi-omics approach combined with pooled mutant fitness assays can be integrated to uncover novel insights into microbial acclimatization to environmental change in non-model systems. Prior studies on acid tolerance in gram-negative bacteria have been largely limited to human symbionts and pathogens (103), neglecting the diversity of tolerance mechanisms that may exist in free-living environmental bacteria, which can also experience wide environmental fluctuations in pH. Understanding these acid tolerance mechanisms in environmental bacteria is important for predicting how pH shifts can impact key microbially mediated environmental processes, such as nitrate removal from contaminated groundwater.

## Data Availability

The generated mass spectrometry proteomics data have been deposited to the ProteomeXchange Consortium via the PRIDE partner repository with the dataset identifier PXD064015 (104). Raw data files for the metabolomics experiments are available in MZML format through https://gnps2.org/status?task=29f23392a1414aa2a75002938e2fecec. DIA-NN is freely available for download from https://github.com/vdemichev/DiaNN.

## SUPPLEMENTAL MATERIAL

Supplemental material aem.01942-25-s0001.docx Supplemental methods, Fig. S1 and S2, and Table S11.

Table S1 aem.01942-25-s0002.xlsx MT123 genome annotations used for this study.

Table S2 aem.01942-25-s0003.xlsx Mapping of RefSeq locus tags to study locus tags.

Tables S3 and S4 aem.01942-25-s0004.xlsx Metabolite annotations and LCMS parameters.

Tables S5-S8 aem.01942-25-s0005.xlsx Proteins significantly increased or decreased in abundance under aerobic or denitrifying growth conditions.

Table S9 aem.01942-25-s0006.xlsx Differentially abundant metabolites at pH 5.5 relative to pH 7.5 under aerobic and denitrifying conditions.

Table S10 aem.01942-25-s0007.xlsx Δfit(pH 5.5-pH 7.5) values calculated from the RB-TnSeq gene fitness data.

ASM does not own the copyrights to Supplemental Material that may be linked to, or accessed through, an article. The authors have granted ASM a non-exclusive, world-wide license to publish the Supplemental Material files. Please contact the corresponding author directly for reuse.
